# Don't take their word for it: Investigating the diagnostic accuracy of history elements for anterior cruciate ligament tears

**DOI:** 10.1002/jeo2.70586

**Published:** 2025-12-07

**Authors:** Alan Price, Rajkumar Gangadharan, Damon Simmons, William Boswell, Thomas W. Maddox, Rachel A. Oldershaw, Richard Norris

**Affiliations:** ^1^ Therapies Department Aintree University Hospital, Liverpool University Hospitals NHS Foundation Trust Liverpool UK; ^2^ Department of Trauma and Orthopaedics Aintree University Hospital, Liverpool University Hospitals NHS Foundation Trust Liverpool UK; ^3^ Radiology Department Aintree University Hospital, Liverpool University Hospitals NHS Foundation Trust Liverpool UK; ^4^ Small Animal Teaching Hospital, Institute of Infection, Veterinary and Ecological Sciences, Faculty of Health and Life Sciences University of Liverpool Neston Wirral UK; ^5^ Department of Musculoskeletal and Ageing Sciences, Institute of Life Course and Medical Sciences, Faculty of Health and Life Sciences University of Liverpool Liverpool UK; ^6^ MRC‐Versus Arthritis Centre for Integrated research into Musculoskeletal Ageing (CIMA), Institute of Life Course and Medical Sciences, Faculty of Health and Life Sciences University of Liverpool Liverpool UK

**Keywords:** anterior cruciate ligament (ACL), history elements, knee injury, predictive validity

## Abstract

**Purpose:**

The primary objective of this study was to investigate the association between patient‐reported history elements and anterior cruciate ligament (ACL) tears. The secondary objectives were to evaluate the predictive validity of history elements and develop clinically interpretable decision rules to aid diagnostic reasoning.

**Methods:**

This was a retrospective analysis of data collected during two prospective studies. Patient history elements including demographics, mechanism of injury, signs/symptoms at the time of injury, and subsequent symptoms since the injury were collected using a pre‐existing departmental questionnaire and cross referenced with magnetic resonance imaging results. Association and predictive validity were investigated through penalised and unpenalised logistic regressions, and classification and regression tree analyses.

**Results:**

Of the 173 included participants, 87 participants (56 males) had an ACL tear with the remainder reported as having posterior cruciate ligament, meniscal, patellofemoral joint, medial and/or lateral knee injuries. There was a significant negative association between seeing deformity (*p* = 0.028, odds ratio [OR]: 0.091, 95% confidence interval [CI]: 0.011–0.766) or isolated anterior knee pain (*p* = 0.038, OR: 0.068, 95% CI: 0.005–0.864) at the time of injury, and an ACL tear. Other patient‐reported history elements, used individually or in combination, demonstrated limited clinical utility for differentiating ACL tears from other traumatic knee injuries.

**Conclusion:**

Patient‐reported deformity and isolated anterior or medial knee pain at the time of injury were significantly associated with the absence of an ACL tear. Other history elements were not significantly associated with an ACL tear, highlighting the limited clinical utility of history taking and the importance of physical examination after knee injury.

**Level of Evidence:**

Level I.

**Abbreviations**
ACL: anterior cruciate ligament
AED: accident and emergency department
AKIC: acute knee injury clinic
AUC: area under the curve
BCa: bias‐corrected and accelerated
CHAMP: CHecklist for statistical Assessment of Medical Papers
CI: confidence interval
C&RT: classification and regression tree
IQR: interquartile range
LASSO: least absolute shrinkage and selection operator
LM: lateral meniscus
LPI: lateral patellar instability
LR+: positive likelihood ratio
LR‐: negative likelihood ratio
MM: medial meniscus
MRI: magnetic resonance imaging
NHS: National Health Service
OR: odds ratio
PCL: posterior cruciate ligament
PFJ: patellofemoral joint
Q‐Q plots: quantile quantile plots
ROC: receiver operator characteristic
Sn: sensitivity
Sp: specificity
SPSS: statistical product and service solutions
STARD: standards for reporting diagnostic accuracy studies

## INTRODUCTION

The incidence of anterior cruciate ligament (ACL) tears is rising, especially in females, with current estimates predicted to be more than double by 2031 [14]. ACL tears can lead to further knee injury and are associated with a 7–8‐fold increase in the odds for developing knee osteoarthritis [22], therefore prompt and accurate diagnosis is critical to mitigate these risks. Magnetic resonance imaging (MRI) is a valid, noninvasive alternative to arthroscopy [19], but the time taken for imaging to be performed, reported, and acted on delays the diagnosis, highlighting the crucial role of clinical evaluation in the early identification of ACL tears.

For experienced assessors, a combination of patient history and clinical examination is often sufficient to diagnose an ACL rupture [9]. When examination is limited (e.g., due to pain, effusion or patient guarding) history‐taking becomes the primary source of diagnostic information. Although distinct situational patterns and mechanisms of ACL injury have been identified in professional sports [11], there is limited evidence for amateur athletes or nonsporting injuries, and similar mechanisms have been associated with knee injuries that do not involve the ACL [4]. A ‘popping’ sensation at the time of injury and rapid swelling are commonly associated with an ACL tear [9], but the supporting evidence demonstrates several methodological limitations, including small sample sizes [8, 10] and the lack of appropriate control groups [1, 7], which could overestimate their diagnostic accuracy.

Determining accurate diagnostic estimates for patient‐reported history elements informs clinical practice and could facilitate the early identification of an ACL tear, especially when physical examination is inconclusive or clinicians are less confident in performing clinical tests. Therefore, the primary objective of this study is to investigate the association between individual and combined history elements and ACL tears. The secondary objectives are to evaluate the predictive validity of history elements and develop clinically interpretable decision rules to aid diagnostic reasoning. We hypothesise that history elements typically associated with ACL injury, including a noncontact change of direction, feeling or hearing a ‘pop’, or knee swelling within 2 h of injury, would not be significantly associated with ACL tears when compared with an injured control group.

## METHODS

### Study design

This was a retrospective analysis of participant data collected during two prospective studies that investigated the diagnostic accuracy of clinical tests for ACL tears (ClinicalTrials.gov: NCT05416632). The study is reported using the Standards for Reporting Diagnostic accuracy studies (STARD) 2015 guidelines [5], and the CHecklist for statistical Assessment of Medical Papers (CHAMP) [15].

### Study population

Patients presenting to the outpatient acute knee injury clinic (AKIC) at Aintree University Hospital were used as the study population. Patients are referred to the AKIC if they present to an accident and emergency department (AED) with a traumatic knee injury, but no clinical or radiographic evidence of fracture, tibiofemoral joint dislocation, or knee extensor tendon rupture.

### Inclusion and exclusion criteria

Data were included for participants aged 18 years or older, who had provided informed written consent, attended the AKIC for clinical assessment, and subsequently underwent MRI examination of their knee. To ensure data were associated with the current presenting injury, participants reporting a previous ipsilateral knee injury or further injury between the AKIC assessment and MRI examination were excluded.

### Procedure

History element data were collected using a standardised knee injury questionnaire (Supporting Information S1: File S1). Consecutive patients presenting to the AKIC during the data collection phase of the relevant studies (November 2022–July 2024) were asked to complete a paper version of the questionnaire in the waiting area immediately before their consultation. During the consultation, the completed questionnaire was reviewed with the patient to ensure they had answered the questions correctly. Patients were asked to provide additional details based on their responses to ensure a comprehensive and accurate history was obtained.

Patients were managed according to their individual presentations with the decision to refer for MRI examination guided by the Trust's clinical pathway (e.g., joint instability, extension deficit/locked knee, suspected unstable meniscal tear). For patients that underwent MRI examination, history elements were cross referenced with the MRI outcome. In the context of this study, an ACL tear was defined as a full or partial‐thickness tear reported on MRI.

### Knee injury questionnaire

The questionnaire was a pre‐existing clinical tool that was developed by AKIC clinicians (physiotherapists and orthopaedic knee consultant surgeons) based on the published literature and clinical experience. To minimise dichotomous forcing bias, open‐ended questions and multiple response options are used where appropriate. History elements collected included patient demographics (age and sex), mechanism of injury (activity, situational pattern), symptoms at the time of injury (pain location, noises/sensations felt or heard), deformity, ability to continue the activity or weight bear, presence and onset of swelling/effusion, and mechanical symptoms since the injury (locking/catching, recurrent knee instability). In total, 14 history elements were collected for analysis, with each category defined in Supporting Information S3: Table S1.

### Assessor

The AKIC assessor was an extended scope physiotherapist with 21 years' clinical experience and 19 years' experience working in an AKIC.

### Reference standard

MRI was used as the reference standard as it is likely to correctly classify the target condition, and the routine use of arthroscopy is not appropriate following traumatic knee injury [18]. All MRI examinations were conducted using a 1.5T or 3.0T Philips Ingenia MR system with all reports verified by a single consultant musculoskeletal radiologist involved in the studies.

### Timing and flow

History elements (index data) were collected before reference testing. The same reference standard was used for all participants, and all participant data were included in the analyses. Data for individual history elements were coded as ‘missing’ if the participant could not provide an accurate response or specific elements were irrelevant.

### Data analysis

Statistical analyses were performed using SPSS Statistics (IBM Corp.; version 30.0) and R (R Foundation for Statistical Computing; version 2025.05.1), with the relevant assumptions considered for each statistical test. Continuous variables were assessed for normality by graphical analysis (histograms and normal Q–Q plots) and normality testing (Kolmogorov–Smirnov and Shapiro–Wilk tests). Parametric or nonparametric tests were used as indicated. Point estimates for statistical tests are reported with 95% confidence intervals (CIs) where appropriate.

### Variable selection

Univariable logistic regression was performed to determine the strength and direction of association between individual history elements and an MRI‐reported ACL tear (dependent variable). A participant‐reported ‘pop’, and ‘pop or snap’ were used as separate elements as both have previously been associated with an ACL tear [10, 20]. Linearity of continuous variables with respect to the logit of the dependent variable (normal or torn ACL) was assessed via the Box‐Tidwell procedure [3].

To identify the history elements most strongly associated with an ACL tear, multivariable logistic regression with Least Absolute Shrinkage and Selection Operator (LASSO) penalisation was performed using the ‘glmnet’ package in R. This approach applies an L1 regularisation penalty that shrinks the coefficients of less informative predictors to zero, thereby excluding them from the model. The optimal regularisation parameter (*λ*) was determined via 10‐fold cross‐validation, and the final model was selected using the minimum cross‐validation error criterion (*λ*_min_).

Variables retained in the LASSO model were subsequently entered into an unpenalised multivariable logistic regression to estimate the strength and direction of association with ACL tears, while adjusting for potential confounders. The final model was bootstrapped with 1000 resamples to evaluate model stability and internal validity, using the ‘boot’ package in R. Odds ratios (ORs) were reported with 95% bias‐corrected and accelerated (BCa) CIs derived from the bootstrap resamples.

### Predictive modelling

To evaluate model performance, the dataset was randomly split into 70% training and 30% test data. The LASSO model was developed on the training set, and predictive performance metrics including sensitivity, specificity, predictive values, likelihood ratios, and the area under the receiver operating characteristic (ROC) curve (AUC) calculated on the test set. AUC values were interpreted as follows: no discrimination (0.5), very poor (0.5–0.6), poor (0.6–0.7), acceptable (0.7–0.8), good (0.8–0.9), excellent (0.9–1.0) and perfect discrimination (1.0) [6].

The same variables were then entered into a classification and regression tree (C&RT) analysis, which recursively partitions the dataset based on the most informative predictors to produce a simple, interpretable decision‐making tool. Node splits were determined using the Gini impurity criterion, with minimum parent and child node sizes set at 15 and 8, respectively. To reduce model complexity and overfitting, cost‐complexity pruning was applied with a pruning value of 1 standard error. Predictive performance was evaluated using ten‐fold cross‐validation, which also guided optimal tree size selection.

## RESULTS

**Figure 1:**
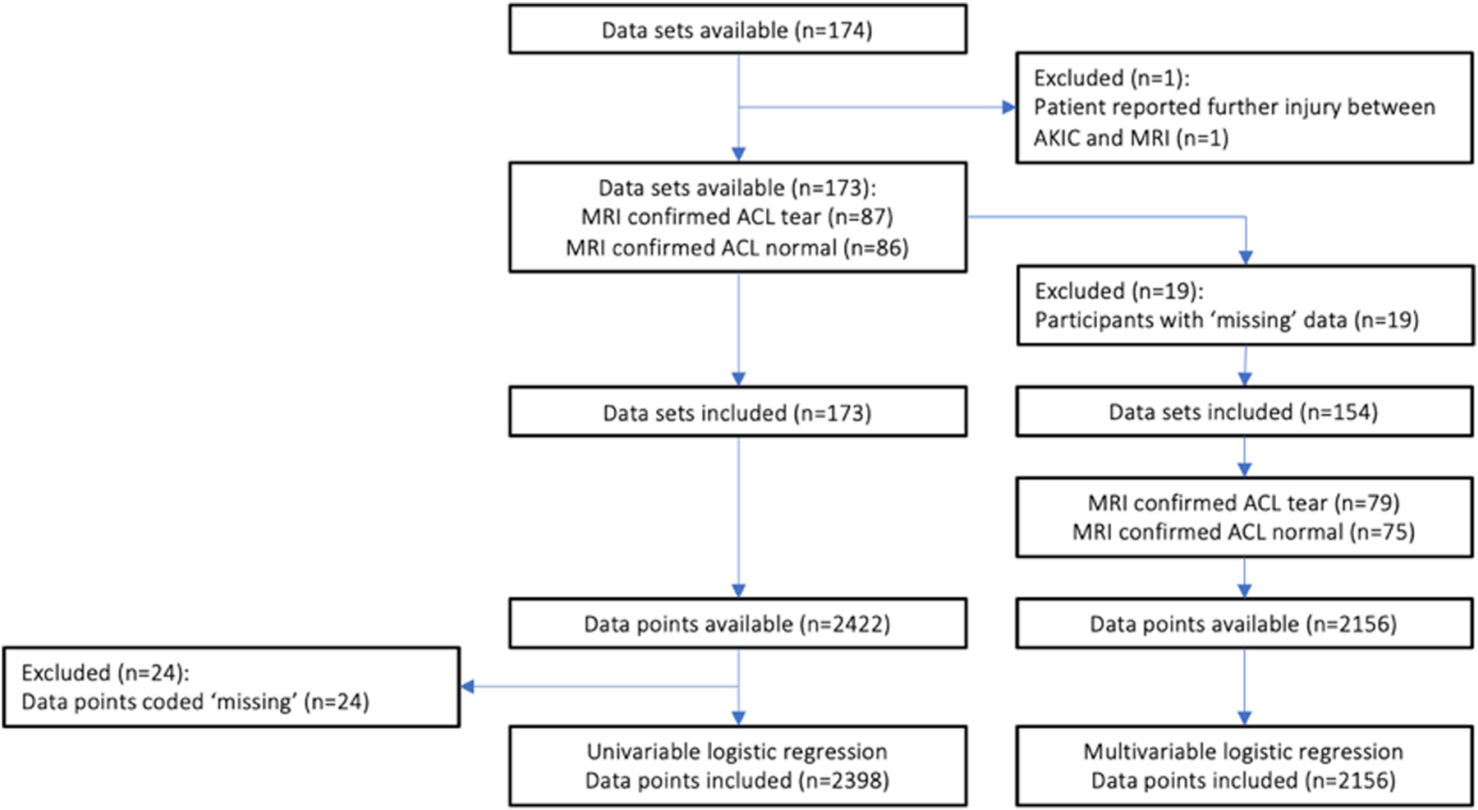
Participant flow. ACL, anterior cruciate ligament; AKIC, acute knee injury clinic; MRI, magnetic resonance imaging.

A total of 174 participant data sets were available, one of which was excluded due to further injury reported between the AKIC and MRI examination (Figure 1). Of the 2422 available data points, 24 (1.0%) were coded ‘missing’ from 19 participants, who were either ‘unsure’ for a specific history element or not performing an activity that they needed to continue (Supporting Information S3: Table S2). Missing data points were not included in the univariable analysis of the respective history element, and the 19 participants with ‘missing’ data were removed from the multivariable analyses.

Based on the MRI reports for the 173 included participants, 74 (42.8%) had a full‐thickness ACL tear (48 males, 26 females) and 13 (7.5%) had a partial‐thickness ACL tear (8 males, 5 females) (Supporting Information S2: File S2). For the 86 participants with a normal ACL, 45 (52.3%) injuries involved the tibiofemoral joint (33 males, 12 females), 34 (39.5%) involved the patellofemoral joint (22 males, 12 females), and 7 MRIs (8.1%) were reported as normal (4 males, 3 females). Only five (5.7%) of the 87 ACL tears occurred without concomitant ligament or meniscal injury. Notably, an ACL tear did not occur concomitantly with a patellar subluxation/dislocation, and patellofemoral joint injuries rarely occurred in combination with tibiofemoral joint injuries (Table 1). No adverse events occurred during the studies (0%).

**Table 1:** Frequency of injuries reported on magnetic resonance imaging.

		Combined with							
Injury (*n*)	Isolated	ACL	PCL	Medial	Lateral	MM	LM	LPI	PFJ
ACL (87)	4 (5%)	‐	7 (8%)	55 (63%)	28 (32%)	42 (48%)	35 (40%)	0 (0%)	1 (1%)
PCL (11)	1 (9%)	7 (64%)	‐	8 (73%)	4 (36%)	6 (55%)	4 (36%)	0 (0%)	0 (0%)
Medial (72)	4 (6%)	58 (77%)	8 (11%)	‐	22 (31%)	30 (42%)	20 (28%)	8 (11%)	1 (1%)
Lateral (31)	2 (6%)	28 (90%)	4 (13%)	22 (71%)	‐	16 (52%)	13 (42%)	0 (0%)	0 (0%)
MM (59)	13(22%)	42 (71%)	6 (10%)	30 (51%)	16 (27%)	‐	21 (36%)	1 (2%)	0 (0%)
LM (45)	7 (16%)	35 (78%)	4 (9%)	20 (44%)	13 (29%)	21 (47%)	‐	0 (0%)	1 (2%)
LPI (28)	20 (71%)	0 (0%)	0 (0%)	8 (29%)	0 (0%)	1 (4%)	0 (0%)	‐	0 (0%)
PFJ (6)	5 (83%)	1 (17%)	0 (0%)	0 (0%)	0 (0%)	0 (0%)	1 (17%)	0 (0%)	‐

*Note*: ‘Isolated’ indicates that no other injuries from the listed categories were present. Medial includes the main medial knee stabilisers (medial collateral ligament and posteromedial corner structures). Lateral includes the main lateral knee stabilisers (lateral collateral ligament and posterolateral corner structures). Table colour coded for co‐occurrence of 0%–10% (green), 11%–30% (yellow), 31%–70% (orange) and 71%–100% (red).

Abbreviations: ACL, anterior cruciate ligament; LM, lateral meniscus; LPI, lateral patellar instability; MM, medial meniscus; PCL, posterior cruciate ligament; PFJ, patellofemoral joint.

### Demographics

The median age was 27 years for both the ACL‐injured (interquartile range [IQR] = 14) and ACL‐normal group (IQR = 12). The median time from injury to assessment was 11 days for both the ACL‐injured (IQR = 10) and ACL‐normal group (IQR = 12). Mann–Whitney *U*‐tests demonstrated no significant difference in age (*p* = 0.678) or time from injury to assessment (*p* = 0.426) between groups.

### Mechanism of injury

For both groups, most injuries were caused during a sporting activity and with a noncontact mechanism (Supporting Information S3: Table S3). Football (soccer) was the most frequently reported activity for the ACL‐injured (47.1%) and ACL‐normal group (36.0%). For the ACL‐injured group, other sporting activities included court sports (netball, badminton, basketball), field sports (American football, rugby union, ultimate frisbee), combat sports (karate, kickboxing, mixed martial arts, Muay Thai, taekwondo), skiing, bouldering and running on an uneven surface. Nonsporting ACL injuries occurred when landing from a height (ladder and stool), slipping (wet surface and sand), dancing and twisting during activities of daily living or when carrying heavy objects (another person and paving stones) (Supporting Information S2: File S2).

### Symptoms at the time of injury

A ‘pop’ was the most frequently reported noise/sensation at the time of injury for both the ACL‐injured (31.0%) and ACL‐normal group (33.7%). For the ACL‐injured group, there were 23 different combinations of noises/sensations and 25 unique pain patterns; 14 (16.1%) individuals reported no noise/sensation and 8 (9.2%) experienced no pain (Supporting Information S1: File S2). The ‘whole knee’ was the most frequently reported area of pain for the ACL‐injured group (26.4%) with 52 (59.8%) participants reporting pain in more than one location.

### Univariable logistic regression

Participant age was linearly related to the logit of the dependent variable. There were four outliers for pain location and two for deformity, which were inspected and kept in the analyses. There was a statistically significant association for pain location (*χ* ^2^ = 25.467 [5], *p* < 0.001) and deformity (*χ* ^2^ = 24.487 [2], *p* < 0.001), with isolated anterior knee pain, isolated medial knee pain, seeing deformity and feeling deformity being negatively associated with an ACL tear. There was no significant association between other history elements and an ACL tear (Supporting Information S3: Table S4).

### Variable selection

The LASSO coefficients for a noncontact mechanism, isolated anterior knee pain, isolated medial knee pain, seeing deformity, feeling deformity, inability to continue and effusion within 2 h of injury were nonzero (Supporting Information S3: Table S5 and Supporting Information S3: Figure S1). All other coefficients were shrunk to zero and the corresponding history elements were therefore excluded from the unpenalised logistic regression model. The model was statistically significant (*χ*² = 34.322 [11], *p* < 0.001) with seeing deformity and isolated anterior knee pain contributing significantly; both history elements were negatively associated with an ACL tear (Table 2). The Hosmer–Lemeshow goodness‐of‐fit test indicated a good fit (*χ*² = 2.509 [7], *p* = 0.926). The AUC was 0.744 (95% CI: 0.667–0.821), indicating acceptable discriminative ability in differentiating ACL tears from other traumatic knee injuries.

**Table 2:** Unpenalised multivariable logistic regression.

				Final model	Bootstrapped model
History element	*B*	*p* value	OR	95% CI for OR	95% BCa CI for OR
Deformity seen	−2.402	0.028*	0.091	0.011–0.766	<0.001–0.34
Deformity felt	−1.636	0.157	0.195	0.020–1.873	<0.001–1.11
Isolated anterior knee pain	−2.683	0.038*	0.068	0.005–0.864	<0.001–0.35
Isolated medial knee pain	− 2.135	0.067	0.118	0.012–1.163	<0.001–0.56
Inability to continue	0.653	0.080	1.922	0.924–3.995	<0.001–2.76 × 10^17^
Noncontact	−0.456	0.373	0.634	0.233–1.728	0.15–1.99
Effusion <2 h	0.394	0.310	1.484	0.692–3.179	0.55–4.04

Abbreviations: BCa, bias‐corrected and accelerated; CI, confidence interval; OR, odds ratio.

* *p* value < 0.05. B: logistic regression coefficient.

### Predictive modelling

The final LASSO and C&RT models both demonstrated limited predictive utility when evaluated on the held‐out test set (Supporting Information S3: Table 6). The AUC for the LASSO model was 0.50 (95% BCa CI: 0.37–0.57), indicating no discriminative ability. The C&RT model identified several key history elements associated with the classification of an ACL tear. The initial split occurred on deformity, where patients who reported seeing or feeling deformity had a lower probability of an ACL tear compared to those who did not. Among individuals without deformity, isolated anterior or medial knee pain at the time of injury was also associated with a reduced likelihood of an ACL tear. Conversely, patients who reported pain in other locations had a 62.3% probability of having an ACL tear (Supporting Information S3: Figure S2).

### Significant history elements

Of the 16 participants that reported seeing deformity, 14 (87.5%) dislocated their patella, one (6.3%) dislocated their tibiofemoral joint, and one (6.3%) had an isolated lateral collateral ligament tear. Using univariable logistic regression, seeing deformity (*p* < 0.001, OR: 140.00, 95% CI: 26.49–739.80) and isolated anterior knee pain (*p* = 0.033, OR: 11.56, 95% CI: 1.22–109.19) were significantly associated with patellar subluxation/dislocation; however, the CIs were wide.

## DISCUSSION

The most important findings from this study are the significant association between deformity, knee pain location at the time of injury, and the absence of an ACL tear. Other patient‐reported history elements, used individually or in combination, were not significantly associated with an ACL tear, therefore the null hypothesis could not be rejected.

Clinical practice guidelines state that an ACL rupture should be suspected if a patient reports a mechanism that involves deceleration/acceleration in combination with a knee valgus load, hearing or feeling a ‘pop’ at the time of injury, or haemarthrosis within 2 h of injury [9]. This poses several challenges for the clinician as patients may not be able to accurately recall their knee position at the time of injury and video evidence is rarely available for amateur sports or nonsporting injuries. Sounds or sensations experienced at the time of injury may also be interpreted differently between individuals. Furthermore, unless the assessment is performed within 2 h of injury, knee effusion within this timeframe relies on the patient's ability to accurately recall the timing and location of swelling.

### Mechanism of injury

In the current study, most ACL tears involved a noncontact mechanism whilst playing football (soccer). However, this was also the most frequently reported mechanism for the ACL‐normal group, suggesting that a change of direction without contact during sports is a common mechanism of knee injury regardless of the involved structures. A pivoting traumatic mechanism has previously been associated with ACL tears; [7] however, the control group appeared to include a substantial proportion of participants without a traumatic injury, which may have inflated the validity estimates.

### ‘Popping’ sensation

Previous studies investigating the association between a reported ‘pop’ or ‘snap’ and ACL injury have collected data using dichotomous yes/no response options [1, 7, 8, 10, 21] and are therefore at risk of dichotomous forcing bias [13]. For example, an individual that experiences a similar sensation or noise at the time of injury may still respond ‘yes’ when asked if there was a ‘pop’ due to the lack of alternative response options. In the current study, individuals were asked to describe any sensations/noises they experienced in their own words. A ‘pop’ was the most frequently reported noise/sensation at the time of injury in both groups, and there were a similar number of ‘pops’ reported between groups. Participants reported no pain, a ‘crack’, or a 'crunch’ more often than a ‘snap’.

### Haemarthrosis

Knee joint swelling within 2 h of injury is suggestive of intra‐articular bleeding [18]. In the current study, 54% of participants reporting a knee effusion within 2 h of injury had an ACL tear and 18% had a patellar dislocation. These findings are consistent with previous studies that used aspiration or arthroscopy to confirm the presence of blood within the knee joint [16, 18], and suggest that the diagnostic accuracy of a haemarthrosis for ACL tears may be no better than chance. A prospective study including 279 participants also found no association between an effusion within 2 h of injury and an ACL tear [7], while other studies have used different timeframes [1, 16, 23] or excluded patellar dislocations [12], making it difficult to compare results directly.

### Clinical utility of history elements

In the largest study to date, a retrospective analysis of history elements extracted from medical records identified no single diagnostic indicator for an ACL tear [23]. However, deformity and pain location were not included as history elements, several elements could not be analysed due to inconsistent or missing data, and it is not clear whether patellar dislocations were included in the control group [23]. Other diagnostic accuracy studies have highlighted the limited benefit of adding history elements to physical examination findings or questioned the concept of a ‘typical’ ACL injury history [1, 7], which is consistent with the current findings.

### Deformity and pain location

In the current study, deformity or isolated anterior knee pain at the time of injury were significantly associated with the absence of an ACL tear and the presence of patellar instability; these two injuries did not occur concomitantly. This is consistent with data collected from 1145 patients presenting to an AED with a traumatic knee haemarthrosis [16], where only 2 (0.3%) of the 599 ACL tears had a concomitant patellar dislocation on MRI examination. The evidence suggests that ACL tears and patellar dislocations are distinct clinical entities that rarely occur simultaneously in a single traumatic event.

A pragmatic interpretation of these findings is that an ACL tear can be confidently ruled out when there is strong clinical suspicion of patellar dislocation or subluxation. In the absence of deformity or isolated anterior knee pain, isolated medial knee pain at the time of injury may also be suggestive of an alternative diagnosis to an ACL tear. Beyond this, history elements appear to provide limited diagnostic value for differentiating ACL tears from other traumatic knee injuries; therefore, the diagnosis of an ACL tear should rely primarily on the findings from the clinical examination.

### Strengths

This is the first study to investigate the association between deformity, pain location and an ACL tear using an injured control group. Data were collected during two prospective studies, with a standardised knee injury questionnaire that mitigated the risk of missing data. Open‐ended questions were used where possible to negate dichotomous forcing bias, questionnaires were checked by the clinician to ensure the history was accurate, and the same reference standard was used for all participants.

The median time from injury to assessment was less than 2 weeks, which provides clinically relevant data at a time point where clinical examination is likely to be most challenging and reduces the risk of recall bias. Previous studies report median times of 25 weeks [23] to 56 months between injury and assessment [10], or have failed to report the average times [7].

### Limitations

This study was a retrospective analysis of data from two prospective studies; therefore, although history elements were collected before reference testing, a sample size was not calculated a priori for the specific analysis undertaken. Nevertheless, the sample size is larger than that of previous prospective studies [8, 10, 12, 21], or includes more ACL‐injured participants, while also incorporating an injured control group [1, 7]. The final model included five predictor variables and 79 ACL tears (events), which exceeds the recommended threshold of 10 events per variable for multivariable modelling [17].

All participants were referred from an AED; therefore, the findings may not be applicable to patients presenting to nonemergency settings. It is important to reiterate that fractures, tibiofemoral dislocations and knee extensor tendon ruptures, which can also result in deformity, are not routinely referred from an AED to the AKIC. Therefore, recommendations from the current study assume that these injuries have already been excluded. Additionally, all participants were required to be eligible for MRI, which may have introduced selection bias.

A traumatic haemarthrosis was defined as knee joint effusion reported to have developed within 2 h of injury [9]. Consistent with other studies, the knee was not aspirated to confirm blood within the joint; therefore, a haemarthrosis was assumed based on participant‐reported timeframes alone. Although knee valgus is associated with an ACL injury, knee position at the time of injury was not investigated in this study, as patients often found it difficult to recall this detail accurately and video analysis was rarely available for confirmation.

### Future direction

Previous studies have employed different means of collecting data, investigated different history elements, or have failed to define specific elements [8, 12, 21]. For example, knee instability could be interpreted as the knee going out of place at the time of injury [1] or recurrent instability since the injury [2]. Likewise, timeframes for the development of knee swelling differ between studies. To improve consistency and comparability, future research would benefit from a standardised data collection tool incorporating clearly defined history elements. This could be developed using similar methodology to a patient‐reported outcome measure, involving patients and professionals from relevant disciplines to ensure relevance, comprehensibility and comprehensiveness, with appropriate instructions and response options [20].

## CONCLUSION

Patient‐reported deformity and isolated anterior or medial knee pain at the time of injury were significantly associated with the absence of an ACL tear. Other history elements were not significantly associated with an ACL tear, highlighting the limited clinical utility of history taking and the importance of physical examination after traumatic knee injury. These findings should be interpreted with caution as fractures, tibiofemoral joint dislocations and knee extensor tendon ruptures, which may also result in deformity, were not routinely included in this cohort.

## AUTHOR CONTRIBUTIONS

All authors contributed to the study conception and design. Material preparation, data collection and analysis were performed by Richard Norris, Alan Price, Thomas Maddox and Rachel Oldershaw. The first draft of the manuscript was written by Alan Price and Richard Norris, and all authors commented on previous versions of the manuscript. All authors read and approved the final manuscript.

## CONFLICT OF INTEREST STATEMENT

The authors declare no conflicts of interest.

## ETHICS STATEMENT

This study was approved by the NHS Health Research Authority, HRA and Health and Care Research Wales (22/NI/0147).

## Supporting information

Supporting information.

Supporting information.

Supporting information.

## Data Availability

The data sets used and/or analysed during the current study are available from the corresponding author on reasonable request.
