# Supplementary material for: Don't take their word for it: Investigating the diagnostic accuracy of history elements for anterior cruciate ligament tears
Source: J Exp Orthop. 2025 Dec 7;12(4):e70586. doi: 10.1002/jeo2.70586 (PMC12682225; doi:10.1002/jeo2.70586)
Supplement: Supplementary file 1 — Supporting information. [file JEO2-12-e70586-s003.pdf]

Please answer all questions below

1. Which knee have you injured? ☐ Left ☐ Right

2. What date did you injure your knee? ___________________________________

3. Did you have a previous issue with this knee? ☐ No. Go to question 4.

   ☐ Yes. How much had it recovered prior to this new injury? Please circle one box.

No recovery	0%	10%	20%	30%	40%	50%	60%	70%	80%	90%	100%	Full recovery

4. What activity were you doing when you injured your knee, e.g., football, netball?

   ____

5. Was there direct contact to your knee?

   ☐ Yes, direct contact to my knee (e.g., fall onto knee, kicked directly on my knee)

   ☐ Contact to another part of my body but not my knee (e.g., shoulder, foot)

   ☐ No contact to my knee or any other part of my body (e.g., twisted knee)

   ☐ Not sure

6. Please draw on the image below to indicate where the pain was **at the time of injury**. If there was no pain, please leave the image blank.

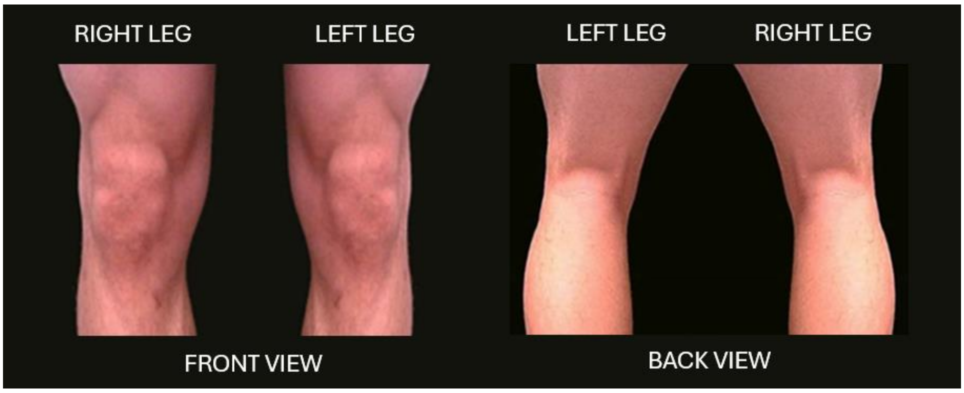

7. How much pain did you have **at the time of injury**? Please circle one box.

No pain	0	1	2	3	4	5	6	7	8	9	10	Worst pain imaginable

8. Did you **see** anything out of position? ☐ No. Go to question 9.

   ☐ Yes (this does not include swelling). What do you think was out of position?

   ____

PLEASE TURN THE PAGE OVER

Please answer all questions below

9. Did you **feel**/**hear** a noise/sensation **at the time of injury** (e.g., crack, pop, snap)?

   ☐ No. Go to question 10. ☐ Yes (this does not include pain)

   If yes, what noise/sensation did you feel? ________________________________

   If yes, what noises did you hear? ____

10. Were you able to continue what you were doing **immediately after the injury**?

    ☐ Yes ☐ No ☐ Tried to but had to stop

11. Were you able to weight bear/walk **immediately after the injury**?

    ☐ Yes, able to take weight through my injured leg (limping is allowed)

    ☐ Unable to take any weight through my injured leg at all

12. Did your knee swell? If it did, how long after the injury did you notice the swelling?

    ☐ Yes, I noticed my knee was swollen within _______________ (minutes, hours)

    ☐ No obvious swelling

    ☐ Not sure

13. Have you been able to fully straighten your knee since the injury?

    ☐ Yes

    ☐ Not initially, but I'm now able to

    ☐ I notice a locking/jamming sensation that can stop my knee from going straight

    ☐ No, I've not been able to fully straighten my knee/lock it out completely

14. Does your knee feel unstable/like it could give way? Tick all boxes that apply.

    ☐ No

    ☐ Yes, with twisting/turning or when changing direction

    ☐ Yes, when moving in a straight line (e.g., walking or using stairs)

    ☐ Yes, but due to muscle weakness rather than the knee joint feeling unstable

15. Please list any medical problems you have and medication you take.

    ____

    ____

16. What do you do for a living/what is your job e.g., student, working, unemployed?

    ____

17. Please list any regular hobbies/sports that you participate in.

    ____

Any other information?
