# Supplementary material for: Don't take their word for it: Investigating the diagnostic accuracy of history elements for anterior cruciate ligament tears
Source: J Exp Orthop. 2025 Dec 7;12(4):e70586. doi: 10.1002/jeo2.70586 (PMC12682225; doi:10.1002/jeo2.70586)
Supplement: Supplementary file 3 — Supporting information. [file JEO2-12-e70586-s002.docx]

| History element | Definition |
| --- | --- |
| Age | Years |
| Sex | Male or female |
| Direct contact | An external force directly applied to the injured knee [6]. |
| Indirect contact | An external force applied to the individual but not directly to the injured knee [6]. |
| Non-contact | Injury occurring without any contact (at the knee or any other level) [6]. |
| Change of direction | Non-contact sporting injury involving deceleration and change of direction. |
| Knee pain location  Isolated  Multiple locations | Extracted from participant markings on anatomical knee images.  Isolated to the anterior, posterior, medial, or lateral aspect of the knee.  Pain in more than one location or if participant reported pain in the ‘whole knee’. |
| Noises/sensations  ‘Pop’  ‘Pop’ or ‘snap’ | Extracted from noises/sensations reported to be felt or heard by the participant.  The participant felt or heard a ‘pop’ +/- other noises/sensations.  The participant felt or heard a ‘pop’ or ‘snap’ +/- other noises/sensations. |
| Knee giving way | Knee going out of position/place and immediately back in [1]. |
| Deformity seen | Participant saw something out of position for a period after injury. |
| Deformity felt | Participant felt something out of position for a period after injury, but did not see it. |
| Ability to weight bear | Able to weight bear 4 steps at the time of injury and in AED; limping is allowed [21]. |
| Ability to continue | Able to complete the activity after injury. |
| Haemarthrosis | Knee effusion that developed within 2 hours of injury [9]. |
| Extension deficit | History of being unable to fully straighten the knee since the injury. |
| Recurrent instability | Further episodes of knee instability since the injury. |

SUPPLEMENTAL TABLE 1: History element definitions

|  | PIN | Coded as ‘missing’ | Reason | Activity | ACL status |
| --- | --- | --- | --- | --- | --- |
| 1 | 72 | Contact | Unsure if contact | Football | Full-thickness tear |
| 2 | 49 | Contact | Unsure if contact | Scooter | Full-thickness tear |
| 3 | 77 | Contact | Unsure if contact | Fall | Partial-thickness tear |
| 4 | 78 | Contact | Unsure if contact | Skiing | Partial-thickness tear |
| 5 | 127 | Contact | Unsure if contact | Football | Normal |
| 6 | 88 | Contact | Unsure if contact | Fight | Normal |
| 7 | 140 | Contact | Unsure if contact | Jiu-jitsu | Normal |
| 8 | 3 | Pain location | Unsure of location | Netball | Full-thickness tear |
| 11 | 103 | ‘Pop’ | Unsure as high velocity | Motorbike | Normal |
| 12 | 103 | ‘Pop’ or ‘snap’ | Unsure as high velocity | Motorbike | Normal |
| 13 | 103 | Knee giving way | Unsure as high velocity | Motorbike | Normal |
| 14 | 4 | Swelling/effusion | Unsure | Football | Full-thickness tear |
| 15 | 37 | Swelling/effusion | Unsure | Skiing | Full-thickness tear |
| 16 | 54 | Swelling/effusion | Unsure | Karate | Full-thickness tear |
| 17 | 134 | Swelling/effusion | Unsure | Football | Normal |
| 18 | 99 | Swelling/effusion | Unsure of timeframe | Slip | Normal |
| 19 | 141 | Swelling/effusion | Unsure of timeframe | Walked into table | Normal |
| 20 | 151 | Swelling/effusion | Unsure of timeframe | Turned in bed | Normal |
| 21 | 101 | Ability to continue | No specific activity | Stood from kneel | Normal |
| 22 | 151 | Ability to continue | No specific activity | Turned on bed | Normal |
| 23 | 144 | Ability to continue | No specific activity | Getting into car | Normal |
| 24 | 154 | Ability to continue | No specific activity | Stood and turned | Normal |

SUPPLEMENTAL TABLE 2: History element data points coded missing

|  | ACL-injured group (n = 87) | | ACL-normal group (n = 86) | |
| --- | --- | --- | --- | --- |
|  | Sports-related  (n = 66) | Non-sporting  (n = 21) | Sports-related  (n = 51) | Non-sporting  (n = 35) |
| Non-contact | 43 (65.2%) | 16 (76.2%) | 37 (72.5%) | 30 (85.7%) |
| Indirect contact | 7 (10.6%) | 0 (0.0%) | 4 (7.8%) | 0 (0.0%) |
| Direct contact | 12 (18.2%) | 3 (14.3%) | 8 (15.7%) | 4 (11.4%) |
| Combined | 2 (3.0%) | 0 (0.0%) | 0 (0.0%) | 0 (0.0%) |
| Unsure | 2 (3.0%) | 2 (9.5%) | 2 (3.9%) | 1 (2.9%) |

SUPPLEMENTAL TABLE 3: Contact mechanism group during sporting and non-sporting activities.

|  | History element | *p* value | OR [95% CI] |
| --- | --- | --- | --- |
| Demographics | Age | 0.707 | 1.005 [0.978 – 1.034] |
|  | Sex | 0.707 | 1.129 [0.600 – 2.123] |
| Mechanism of injury | Change of direction | 0.139 | 1.587 [0.861 – 2.926] |
|  | Non-contact | 0.254 | 0.622 [0.274 – 1.408] |
|  | Indirect contact | 0.773 | 1.235 [0.295 – 5.181] |
| Symptoms at time of injury | Isolated anterior knee pain | 0.001* | 0.020 [0.002 – 0.220] |
|  | Isolated posterior knee pain | 0.532 | 0.375 [0.017 – 8.103] |
|  | Isolated medial knee pain | 0.017* | 0.069 [0.008 – 0.624] |
|  | Isolated lateral knee pain | 0.093 | 0.141 [0.014 – 1.384] |
|  | > 1 location of knee pain | 0.108 | 0.176 [0.021 – 1.465] |
|  | Knee giving way/out of place | 0.415 | 0.740 [0.359 – 1.526] |
|  | ‘Pop’ felt or heard | 0.632 | 1.158 [0.634 – 2.116] |
|  | ‘Pop or ‘snap’ felt or heard | 0.361 | 1.322 [0.726 – 2.407] |
| Symptoms after injury | Deformity seen | 0.004* | 0.049 [0.006 – 0.378] |
|  | Deformity felt | 0.026* | 0.091 [0.011 – 0.748] |
|  | Inability to weight bear | 0.197 | 1.483 [0.814 – 2.700] |
|  | Inability to continue activity | 0.120 | 5.584 [0.638 – 48.858] |
|  | Effusion < 2 hours | 0.210 | 1.510 [0.793 – 2.878] |
|  | Extension deficit | 0.583 | 0.845 [0.462 – 1.544] |
|  | Recurrent instability | 0.494 | 1.237 [0.672 – 2.280] |

SUPPLEMENTAL TABLE 4: Univariable logistic regression values for individual history elements. *Indicates a *p* value < 0.05.

|  | λ_min_ | λ_1se_ |
| --- | --- | --- |
| Non-contact | -0.18773956 | - |
| Isolated anterior knee pain | -0.58188963 | -0.10598275 |
| Isolated medial knee pain | -0.30687212 | - |
| Deformity seen | -1.18923020 | -0.42247422 |
| Deformity felt | -0.65323130 | - |
| Inability to continue | 0.65914745 | - |
| Haemarthrosis | 0.05262206 | - |

SUPPLEMENTAL TABLE 5: Non-zero LASSO logistic regression coefficients using minimum cross validation Lamba (λ_min_) and 1 standard error of the minimum (λ_1se_) thresholds. Negative values indicate an inverse association with ACL tears.

|  | LASSO model [95% BCa CIs] | C&RT analysis [95% CIs] |
| --- | --- | --- |
| Accuracy | 0.57 [0.39 - 0.67] | 0.60 [0.38 – 0.67] |
| Sensitivity | 0.67 [0.46 - 0.82] | 0.74 [0.30 – 0.87] |
| Specificity | 0.42 [0.20 - 0.63] | 0.46 [0.18 – 0.73] |
| Positive PV | 0.62[ 0.40 - 0.77] | 0.59 [0.39 – 0.67] |
| Negative PV | 0.47 [0.21 - 0.71] | 0.63 [0.35 – 0.71] |
| Positive LR | 1.15 [0.75 - 2.02] | 1.36 [0.62 – 1.91] |
| Negative LR | 0.79 [0.35 - 1.77] | 0.57 [0.38 – 1.79] |
| AUC | 0.50 [0.37 - 0.57] | 0.52 [0.45 - 0.68] |

Table 6: Predictive performance metrics. AUC: area under the curve; BCa: Bias-Corrected and Accelerated; CIs: confidence intervals; C&RT: Classification and Regression Tree; LASSO: Least Absolute Shrinkage and Selection Operator; LR: likelihood ratio, PV: predictive value.


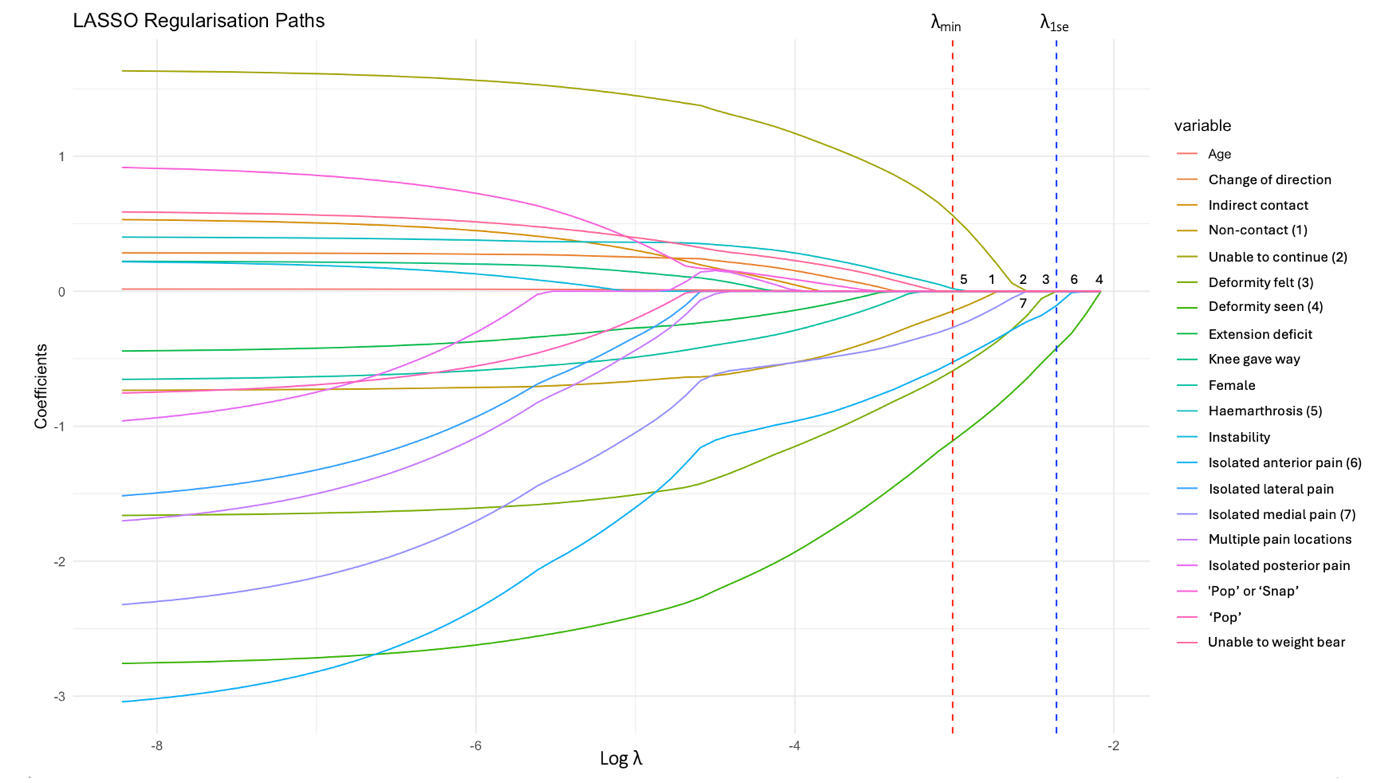


SUPPLEMENTAL FIGURE 1: LASSO regularisation path plots for history elements, with the minimum cross validation Lamba (λ_min_) and 1 standard error of the minimum (λ_1se_) indicated by the red and blue vertical dotted lines.


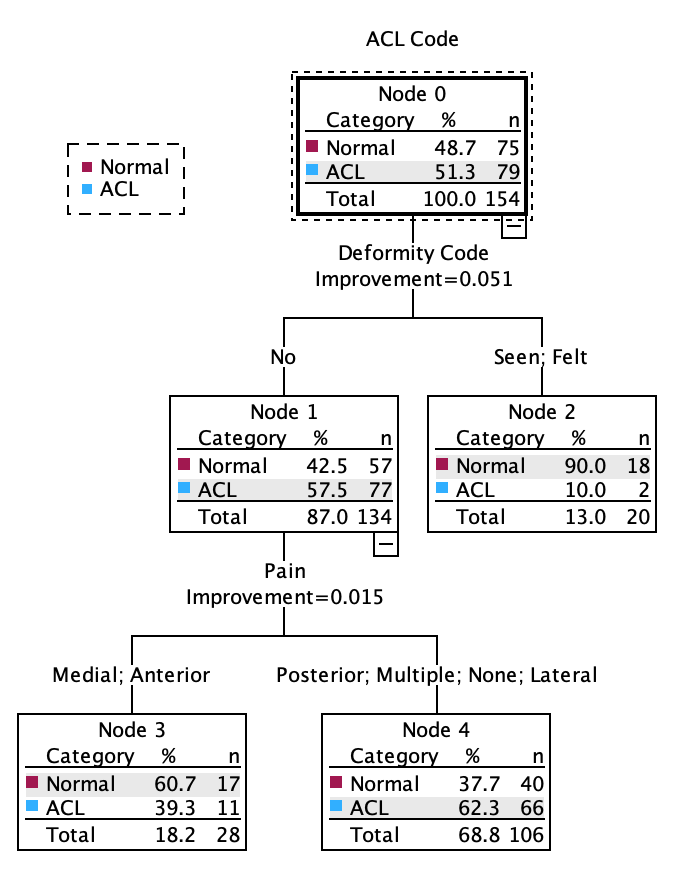


SUPPLEMENTAL FIGURE 2: Classification and Regression Tree (C&RT) analysis.
